# Transcriptomic Alterations in Water Flea (*Daphnia magna*) following Pravastatin Treatments: Insect Hormone Biosynthesis and Energy Metabolism

**DOI:** 10.3390/toxics10030110

**Published:** 2022-02-25

**Authors:** Yuan Lei, Jiahua Guo, Qiqi Chen, Jiezhang Mo, Yulu Tian, Hisato Iwata, Jinxi Song

**Affiliations:** 1 Shaanxi Key Laboratory of Earth Surface System and Environmental Carrying Capacity, College of Urban and Environmental Sciences, Northwest University, Xi’an 710127, China; 201920855@stumail.nwu.edu.cn (Y.L.); chenqiqi@stumail.nwu.edu.cn (Q.C.); tianyulu@nwu.edu.cn (Y.T.); 2 State Key Laboratory of Marine Pollution, Department of Chemistry, City University of Hong Kong, Kowloon, Hong Kong SAR, China; jiezmo2-c@my.cityu.edu.hk; 3 Center for Marine Environmental Studies, Ehime University, Bunkyo-cho 2-5, Matsuyama 790-8577, Ehime Prefecture, Japan; iwata.hisato.mz@ehime-u.ac.jp

**Keywords:** statins, body length, reproductive toxicity, juvenile hormone, methyl farnesoate

## Abstract

Pravastatin, used for lowering cholesterol and further decreasing blood lipid, has been frequently detected in the contaminated freshwaters, whereas its long-term exposure effects on non-target aquatic invertebrates remains undetermined. Therefore, the purpose of this study was to evaluate the toxic effects of pravastatin (PRA) with the concentration gradients (0, 0.5, 50, 5000 μg/L) on a model water flea *Daphnia magna* (*D. magna*) over 21 d based on phenotypic and genome-wide transcriptomic analyses. After 21 d, exposure to PRA at 5000 μg/L significantly reduced the body length and increased the number of offspring. The 76, 167, and 499 differentially expressed genes (DEGs) were identified by using absolute log2 fold change < 1 and adj *p* < 0.05 as a cutoff in the 0.5, 50, and 5000 μg/L PRA treatment groups, respectively. Three pathways, including xenobiotic metabolism, insect hormone biosynthesis pathway, and energy metabolism were significantly (*p* < 0.05) enriched after exposure to PRA. These suggested that the upregulation of genes in insect biosynthetic hormone pathway increased the juvenile hormone III content, which further reduced the body length of *D. magna*. The positive effect of methyl farnesoate synthesis on the ovarian may result in the increased number of offspring. Furthermore, energy tended to be allocated to detoxification process and survival under stress conditions, as the amount of energy that an individual can invest in maintenance and growth is limited. Taken together, our results unraveled the toxic mechanism of cardiovascular and lipid pharmaceuticals in aquatic invertebrate.

## 1. Introduction

Statins act as cholesterol-lowering drugs that inhibit HMG-CoA reductase through binding to the enzyme’s active site, which block the substrate product transition state of the enzyme in humans [1,2]. Contrary to atorvastatin and simvastatin, pravastatin (PRA) possesses the highest stability and accounts for 25% of statin use on the market [3]. However, PRA entering the body cannot be fully absorbed and metabolized. For example, approximately 70% of the administered dose of PRA is excreted in faeces and 20% in urine [4]. Since conventional sewage treatment plants are not designed to remove these emerging contaminants, the unaltered statins in effluent are released into the aquatic environment [5,6]. Occurrence of statins (e.g., simvastatin) has been reported in surface water with the maximum concentration of 1.23 μg/L [7]. Concerns over the potentially adverse effects of statins on the aquatic ecosystem have been raised, especially for invertebrates with significant roles in the food chain [8].

Several studies have reported the ecotoxicity of statins to aquatic invertebrates in indoor experiments [9,10,11]. For example, the 96-h LC50 of simvastatin on larval *Palaemonetes pugio* is 1.18 mg/L [9], and the 48-h EC50s of atorvastatin on *Chironomus Tentans* and *Hyalella Azteca* are 1.5 mg/L and 14.3 mg/L, respectively [12]. Energy allocation in organisms, including the water flea, is more inclined to detoxification and survival under stress conditions [13]. Since the amount of energy that an individual can invest in maintenance and growth is limited, increasing energy demands for survival may result in less energy available for growth [14]. For example, *Daphnia magna* (*D. magna*) exposed to 50 μg/L simvastatin leads to downregulation of detoxification related genes (*cyp314*, *ecdysone receptor (ecr)*, and *vitellogenin (vtg*)), which are associated with a reduction in total number of broods per individual, the total egg production number per individual and intrinsic growth rate [7]. *Gammarus locusta* exposed to simvastatin at 320 ng/L for 36 d severely reduced body length and decreased the number of newborns [10]. Simvastain and pravastatin belong to the statins that are used to lower cholesterol and blood lipid. At present, almost all studies on the biological toxicity of statins to the environment focus on simvastatin; however, the ecotoxicity of pravastatin in invertebrates and its mechanism of toxicity remains largely unexplored.

*D. magna* is an ecotoxicological model organism recommended by the Organisation for Economic Co-operation and Development (OECD), owing to its short reproductive cycle, parthenogenesis, and its easy manipulation in laboratory conditions, and high sensitivity to toxicants [15,16,17]. Notably, compared with other crustacean species, the genome of *D. magna* has been fully sequenced, which enables a genome-wide investigation on the transcriptional level in response to chemical stressors [18,19]. Quantitative real-time PCR analysis has been applied to decipher the potential molecular mechanism of *D. magna* in response to simvastatin [7]. It is suggested that detoxification of simvastatin consumes a large amount of energy, which further reduces the energy allocated to growth and reproduction of *D. magna* [7]. In general, quantitative real-time PCR only analyzes the expression level of a few genes; thus, the genome-wide transcriptional alterations and full extent of dysregulated pathways in *D. magna* exposed to simvastatin remain largely unknown. The measurement of gene expression on a genome-wide scale can be achieved using next-generation sequencing (NGS).

The purpose of this study was to evaluate the effects of pravastatin (0, 0.5, 50, 5000 μg/L) on the growth and reproductive fitness of *D. magna* after 21 d of long-term exposure and to elucidate its underlying toxic mechanism using transcriptomic analysis. We considered 0.5 μg/L to be an environmentally relevant concentration; the effects of PRA on the environment were investigated. We determined that 5000 μg/L was approximately half of EC50 for 48 h obtained in the preliminary test. In addition, the remaining high concentrations were used to explore the dose–response effect for revealing the mechanism of toxicity. It was hypothesized that exposure to PRA interferes with the signaling pathways in relation to juvenile hormone biosynthesis, energy metabolism, and the typical reproduction gene (*cyp314*, *ecr*, and *vtg*), which ultimately inhibits body length and changes reproductive strategies of *D. magna*.

## 2. Materials and Methods

### 2.1. Chemical Reagents

Pravastatin sodium (HPLC ≥ 98.0%, CAS no. 81131-70-6) was purchased from Yuanye Biotechnology Co., Ltd. (Shanghai, China). All chemicals used for preparation of Elendt M4 (pH 7.76–7.84) and ISO test water (pH 7.76–7.84) were at least reagent grade [15], which were used for culture and test, respectively.

### 2.2. Biotests and Culture Conditions

*D. magna*, obtained from Guangdong Laboratory Animals Monitoring Institute, was cultured in Elendt M4 (pH 7.76–7.84); its culture condition in our laboratory was according to OECD guidelines 202 [15]. To ensure that *D. magna* (age < 24 h) used in all experiments were the third brood of the parent animals, 50 neonates (age < 24 h) were cultured in 2 L medium as the parent animals, and the medium was changed every two days. *D. magna* (age < 24 h) derived from the parent animals were fed with green alga *Chlorella Vulgaris* (0.1–0.2 mg total organic carbon (TOC) per *D. magna*) every two days, *Chlorella Vulgaris* (FACHB-8) was purchased from Freshwater Algae Culture Collection, Institute of Hydrobiology, Chinese Academy of Sciences (Wuhan, China). They were cultivated with controlled light intensity (1500 Lux), temperature (22 ± 1 °C) and photoperiod (light:dark = 16 h:8 h).

### 2.3. D. magna Immobilization Study

The acute test was conducted in accordance with the requirements of OECD Guidelines 202 [15]. PRA was dissolved in ISO test water and ultrasonically treated for 1 h to prepare PRA stock solution with a concentration of 25 mg/L, followed by preparing nine test concentrations (0, 2.65 mg/L, 5.3 mg/L, 7.0 mg/L, 8.0 mg/L, 10.6 mg/L, 12.0 mg/L, 18.0 mg/L, 25.0 mg/L) to estimate the EC50; all treatments were in eight parallels; each parallel was prepared with 5 individuals in 200 mL beaker containing 100 mL culture medium (ISO test water). There was no food in the culture medium. The acute toxicity test lasted for 48 h. During this period, no feeding was given. All beakers were checked for immobilization of *D. magna* after 48 h.

### 2.4. Chronic Toxicity Test

The chronic toxicity test was carried out according to OECD Guidelines 211 [20]. In general, there were five clutches in *D. magna* for 21 days, which helped to observe the effect of PRA on offspring of *D. magna*. Meanwhile, following OECD 211, we aimed to compare the experimental results with those of others. Briefly, pravastatin sodium was dissolved in ISO test water to prepare 10 mg /L PRA solution. A control and three exposure concentrations including 0.5 μg/L, 50 μg/L, and 5000 μg/L PRA were used for chronic test. Sixty replicates were prepared for all groups; every replicate contained 20 mL culture medium in a 50 mL beaker. During the experiment, the ISO test water was reprepared every two days and all *D. magna* from the experimental group were moved to the corresponding test medium. Food was also added with 0.1 to 0.2 mg of TOC once every two days, when the culture medium was changed. The body length of each individual was observed weekly. The newborn of parent *D. magna* were counted daily and removed. Other phenotypic parameters, including the number of survivors, molts, and clutches were also recorded.

### 2.5. RNA Sequencing

Firstly, RNA in *D. magna* was extracted by TIANDZ RNA extraction kit (Tiandz, Inc., Beijing, China) following the protocol, and then Nano Drop 2000 (Thermo Scientific, Waltham, MA, USA) was used to determine the amount of RNA extracted in each group, followed by evaluating the RNA integrity by making use of electrophoresis and Agilent 2100 Bioanalyzer (Agilent Technologies, Inc., Santa Clara, CA, USA). After that, all samples were analyzed by NGS using the Illumina Nova Seq platform in Shanghai Personal Biotechnology Corporation (Shanghai, China). During the RNA sequencing process, the numbers of mapped reads ranged from 38,483,716 to 45,913,232, with the coverages of 96.82–97.26%.

### 2.6. RNA-Seq Data Analyses

Raw Data were firstly sorted into FASTQ format for data filtering. Cutadapt (Vision 1.1) software [21] was used to remove the 3′ end joints and reads (QV < 20). Secondly, the filtered data were aligned to the reference genome sequence *the D. magna* 1.0 (GCA-003990815.1) by Hisat2 software (V.2.1.0) [22]. The HTSeq (v. 0.11.1) was used to compare the read count value on all genes as the original expression level of the genes [23]. For the sake of making the gene expression levels of different genes and different samples comparable, FPKM (fragments per kilobases per million fragments) were calculated for normalization [24].

This study performed Pearson’s correlation analysis to check the correlation of gene expression levels among samples. Then, we applied principal component analysis (PCA) with DESeq software package in R software for the sake of reducing the complexity of data. The adj *p* values < 0.05 and absolute log2 fold change > 1 were chosen as a threshold to identify differentially expressed genes (DEGs). Moreover, the volcano map and clustering heat map were constructed by “ggplots2” and “Pheatmap”. Venn diagram was plotted to illustrate the number of intersecting DEG among three PRA treatment groups. Subsequently, two functional enrichment analyses, including gene ontology (GO) and Kyoto Encyclopedia of Genes and Genomes (KEGG) pathways were performed using DEGs.

There were three categories, including molecular functions (MF), biological process (BP), and cellular components (CC) in GO functional enrichment analyzed by “TopGO” package. A *p* value of less than 0.05 was considered as being significantly enriched for KEGG pathway and GO terms.

### 2.7. Quantitative Real-Time Polymerase Chain Reaction (qRT-PCR) Analysis

To confirm the results from NGS, the expression of three genes corresponding to methyl farnesoate epoxidase (*cyp15a1_c1*), glutathione s-transferase (*gst*), dehydrogenase/reductase sdr family member 4 (*dhrs4*) in relation to insect hormone biosynthesis, glutathione metabolism, and retinol metabolism, respectively, were selected for qRT-PCR analysis. One housekeeping gene has been reported and accepted [25]; actin beta/gamma 1 (*actb_g1*) was a housekeeping gene in this study.

Real-time PCR (TIB8600, Triplex International Biosciences, Xiamen, China) was used for relative quantification of gene expression. In the first step, qualified and quantified total RNA was reverse transcribed into cDNA using the PrimeScript ^TM^ 1st STAND cDNA Synthesis Kit. Gene specific QRT-PCR primers in daphnia magna were used for mRNA quantification as follows: *actb_g1-F*: 5′-TGAGCGCAAATACTCCGTCT-3′, *actb_g1-R*: 5′-CCATCGGAAAGCGCCAGAT-3′, length of 152 bp; *cyp15a1_c1-F*: 5′-ACCTAACGGAAGAAGACGCTGATAA-3′, *cyp15a1_c1*-R: 5′-TCAGATGAATAATGGCGAACCCT-3′, length of 183 bp; *gst*-F: 5′-GCGAATGGTTGAGCGAGAA-3′, *gst*-R: 5′-TATCAAGTGCCTCTGCTTCCA-3′, length of 189 bp, *dhrs4*-F: 5′-CGCAGGTGTCAAGGAAGAT-3′, *dhrs4*-R: 5′-CTGGATTAACGGCAACATTCG-3′, length of 98 bp. These four primers were synthesized by Shanghai Personal Biotechnology Co., Ltd. (China). In the second step, for each target gene and housekeeping gene, the cDNA template of the gene was selected for PCR. Each 20 μL reaction mix included 10 μL 2 × SYBR real-time PCR premixture, 0.4 μL forward primer (10 μM), 0.4 μL reverse primer (10 μM), 1 μL cDNA, and 8.2 μL RNase free dH_2_O. The reaction conditions were: 95 °C for 5 min, 95 °C for 15 s, 60 °C for 30 s, a total of 40 cycles. The temperature range was 60 °C~95 °C. Melt curve analysis was performed at the end of each operation. The mRNA levels for each target gene were calculated with the 2^−ΔΔCt^ method and normalized to a housekeeping gene *actb_g1*. For each gene, expression of PRA-treated samples relative to that of controls was estimated.

### 2.8. Statistical Analysis

Statistical analysis on all phenotypic results was conducted using Graph Pad Prism software (version 8.2). To analyze the statistical difference of phenotypes between control and treatment groups, a normality test was initially conducted. If the data failed to pass normality test, a nonparametric test (Kruskal–Wallis test) followed by Dunns post hoc test was performed, otherwise a one-way ANOVA followed by Dunnett’s post hoc test was applied. A *p* value < 0.05 was considered as statistical significance. Besides, a Pearson’s correlation analysis was performed to explore the relationship between qRT-PCR and NGS results.

## 3. Results

### 3.1. Effects of Acute and Chronic Exposures to PRA

**Figure 1:**
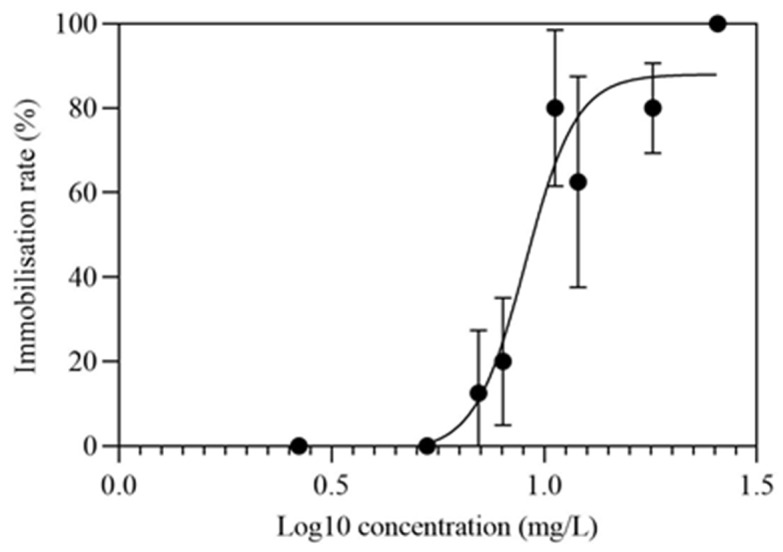
Inhibition rate of *D. magna* exposed to different PRA concentrations for 48 h (*n* = 8).

**Figure 2:**
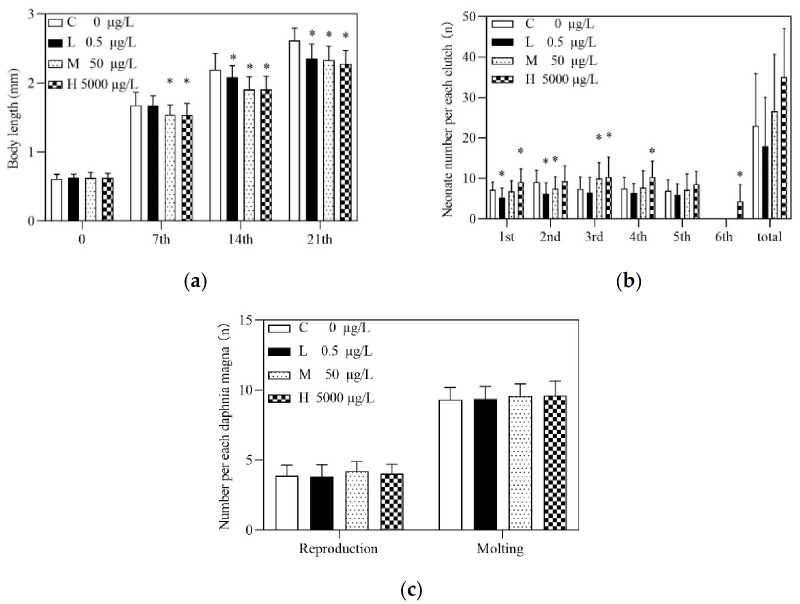
Growth parameters in *D. magna* after 21 d exposure to pravastatin sodium (PRA): (**a**) Body length growth in *D. magna* during 21 d test; (**b**) Offspring per each clutch in *D. magna* during 21d PRA exposure test; (**c**) The average number of reproduction (left) and molting (right) of each *D. magna* during 21 d PRA test. C, L, M and H represent the control, low, medium and high PRA treatment groups, respectively. An asterisk (*) indicates that the parameters of PRA treatment groups are significantly different from control (*p* < 0.05; *n* = 60).

In the immobilization test, a concentration–response curve was plotted to obtain a EC50 value of 9.032 mg/L after 48-h, with the 95% confidence intervals ranging from 8.370 mg/L to 9.817 mg/L (Figure 1). As for chronic toxicity evaluation, the survival of *D. magna* in both the control and three treatment groups in this experiment were 85%, 81.7%, 85%, and 88.3%, respectively. According to OECD 211, maternal mortality of no more than 20% in chronic toxicity tests was normal, whereas the number of mortalities between the treatment group and the control group was considered to be undifferentiated. According to the research, body length was also measured every 7 days [26]. Compared with the control group, the body length of the medium and high treatment groups was significantly reduced after PRA exposure for 7 days. With the extension of exposure time, the body length of *D. magna* in all treatment groups was significantly different from that in the control group. Especially, at day 21, the reduction rates for body length of *D. magna* in 0.5 μg/L, 50 μg/L, and 5000 μg/L PRA were 13.97%, 15.01% and 16.44%, respectively (Figure 2a). However, deformities of the *D. magna* have not been observed.

Regarding the reproductive effects, the total number of offspring was reduced in the low treatment group in comparison to the control, whereas it increased in medium and high groups, though statistical difference was not detected (Figure 2b). Notably, there were five clutches in the control, low and medium PRA treatment group, while there were six clutches in the high PRA treatment group. The number of offspring per clutch under high concentration was always higher than that in the control group. Briefly, significant differences were detected in the first, third, fourth and sixth clutch, as shown in Figure 2b. There was no significant difference between the 50 μg/L PRA and the control groups. As for the low treatment group, the total number of offspring decreased, and the number of offspring per clutch was lower than that in the control group. Meanwhile, no significant difference was found for the number of molts and reproduction between PRA treatment and control groups (Figure 2c).

### 3.2. Transcriptome Analysis

#### 3.2.1. Sequence Assembly and Generation

There were 43,726,361, 43,431,852, 40,943,921 and 39,705,239 raw reads obtained in the control, low, medium and high treatment groups, respectively. Moreover, the Q20 per each sample was above 96.8%. After trimming and quality checking, 40,869,750, 40,414,541, 38,106,117, and 36,934,997 clean reads were retained in these groups; an average of 92.8% clean reads were successfully aligned with the reference genome.

#### 3.2.2. RNA-Seq Reads and DEGs

**Figure 3:**
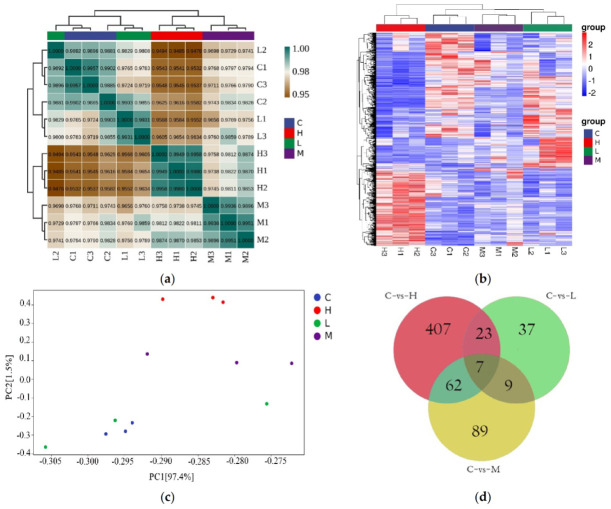
Transcriptomic profiling of *D. magna* following a 21 d exposure to pravastatin sodium (PRA): (**a**) Correlation analysis of patterns of gene expression level in control and three treatment groups; (**b**) A heatmap of centered and scaled FPKM values of DEGs in controls and three treatment groups; (**c**) Principal component analysis (PCA) of FPKM profiles in three treatment groups; (**d**) Venn diagram of the number of DEGs in three treatment groups. L, M and H represent the low, medium and high PRA treatment groups, respectively.

There were 15,351 transcripts totally identified in *D. magna*. Regarding the gene expression patterns, the correlation coefficient between three replicates in control and three treatment groups was not less than 0.98 (Figure 3a). Meanwhile, PCA also showed that gene expression patterns in medium and high groups were differentiable from that in control, whereas the control and the 0.5 μg/L PRA treatment group were closer (Figure 3c). This was in accordance with the results illustrated in heatmap (Figure 3b).

The 76 (52 up-regulated and 24 down-regulated), 167 (45 up-regulated and 122 down-regulated) and 499 (226 up-regulated and 273 down-regulated) DEGs were identified in low, medium and high PRA treatment groups, respectively (Figure 3d and Figure S1). To verify the results of NGS, expression levels of three DEGs related to three pathways, including insect hormone biosynthesis pathway, glutathione metabolism, and retinol metabolism were detected by qRT-PCR. There was a high correlation between the FPKM values and the mRNA expression levels measured by qRT-PCR (Figure S2). For example, the correlation between RNA-seq of genes *dhrs4* and fold changes in mRNA levels determined by qRT-PCR was approximately 0.96. This suggested that the mRNA expression profile provided by NGS was reliable.

#### 3.2.3. Go Ontology and KEGG Pathway Analysis

The GO analysis aimed to investigate the roles of PRA-affected genes in the CC, MF, and BP. The top 10 remarkably enriched GO terms in respect of CC, MF, and BP enriched in the low, medium, and high ERY treatment groups were summarized in Supplementary Materials (Figure S3).

Briefly, several GO terms were associated with glucuronate metabolic (GO:0006750 glutathione biosynthetic process, GO:0015020 glucuronosyltransferase activity, GO:0019585 glucuronate metabolic process, etc.) in the low PRA treatment. A few GO terms were related to detoxification (e.g., GO:0006979 response to oxidative stress, GO:1990748 cellular detoxification) in the medium PRA treatment. DEGs in the high PRA treatment were mostly enriched in the GO terms with regard to chitin metabolic process and response to pharmaceutical (e.g., GO:0008236 serine-type peptidase activity, GO:0017171 serine hydrolase activity, GO:0006030 chitin metabolic process, GO:0042302 structural constituent of cuticle, GO:0008233 peptidase activity, GO:0015893 drug transport, GO:0042493 response to drug, GO:0097237 cellular response to toxic substance). Thus, these GO terms were suggested to play crucial roles in the PRA exposed to *D. magna* growth.

Regarding the enriched pathways, PRA exposure interfered with pathways related to insect hormones biosynthesis, protein digestion and absorption, pancreatic secretion and glutathione metabolism (Table 1). Other pathways related to fundamental biological processes such as ECM-receptor interaction and retinol metabolism were shown in Supplementary Materials (Table S1).

## 4. Discussion

The present study unraveled the molecular mechanism of PRA toxicity in *D. magna*. In accordance with our hypothesis, the energy metabolism related pathways such as protein digestion and absorption and pancreatic secretion were suppressed in *D. magna* treated with 5000 μg/L PRA, while insect hormone biosynthesis involved in the reproductive process was up-regulated in all PRA groups (Table 1). After a 21d exposure to PRA, except for reduced body length of *D. magna* and increased reproductive offspring number per individual by 5000 μg/L, no more obvious malformation was found. These phenotypic effects were likely due to the suppressed protein digestion and absorption and decreased pancreatic secretion, and the activated juvenile hormone synthesis. The findings are discussed in the following sections.

### 4.1. Genes Related to Xenobiotic Metabolism

In invertebrates, xenobiotics may be detoxified via phases I, II & III metabolism [27]. In the present study, cytochrome P450 family 4 (*cyp4*) involved in phase I hydrolysis reaction and catalytic single oxidation/hydroxylation reaction was significantly down-regulated with the increase in PRA concentration. However, the phase II glucosylation and methylation genes, including glutathione s-transferase (*gst*), glutathione peroxidase (*gpx*), prostaglandin-h2 d-isomerase/glutathione transferase (*hpgds*), sulfotransferase family cytosolic 1B member 1 (*sult*), and glucuronosyltransferase (*ugt*) were gradually up-regulated in the medium and high PRA treatment groups. This suggested that the phase II was the primary process for metabolizing PRA in *D. magna*. In line with this, *cyp314* and *cyp360a8* were down-regulated, while *gst* was up-regulated in the simvastatin treated *D. magna* [7]. In phase III, ATP-binding cassette transporters (ABC transporters) in organisms were used to remove phase II products to the extracellular medium, where they may be further metabolized or excreted [28]. However, no DEG related to phase III detoxification was identified, suggesting that the phase III detoxification may play a minor role in PRA metabolism in *D. magna*. Under PRA stress of the prolonged presence, energy consumption by exogenous metabolism may lower the allocation of energy for growth and reproduction of *D. magna*, possibly leading to reduction in body length and adjustment of reproductive strategy.

PRA exposure may result in the production of reactive oxygen species (ROS) in *D. magna*, which can damage DNA, proteins, lipids and other biomolecules, accelerating cell senescence and even cell death [29]. However, *D. magna* can metabolize ROS and repair the damage by non-enzymatic glutathione (GSH). In the present study, glutamate–cysteine ligase catalytic subunit (*gclc*) and glutathione synthase (*gshb*) promoted glutathione synthesis. In addition, glutathione peroxidase (*gpx*) in the high PRA treatment group was up-regulated, which may have promoted glutathione synthesis and increased its capacity to catalyze the reduction of harmful hydroperoxides [30]. Therefore, the upregulation of these three genes suggested that PRA at high exposure level may cause oxidative stress in *D. magna*. In addition, the upregulation of *gst* suggested that the enzyme GST catalyzed an enhanced reaction between organic peroxides and enzyme GSH, thus, alleviating ROS [31]. Similar to PRA, simvastatin was shown to induce oxidative stress in *D. magna*, where the enzyme GST increased significantly at 50 μg/L [7].

### 4.2. Genes Related to Insect Hormone Biosynthesis

Metamorphosis development in insects is co-regulated by ecdysone and juvenile hormone synthesis processes [32,33]. Ecdysone regulates larval ecdysis and metamorphosis, whereas juvenile hormones maintain the larval morphology during larval ecdysis by antagonizing ecdysone, thus, preventing metamorphosis from occurring in advance [34,35]. Meanwhile, methyl farnesoate is an isoprenoid equivalent to the insect juvenile hormone [36]. In this study, methyl farnesoate epoxidase (*cyp15a1_c1*) was up-regulated in all PRA treatment groups, and expression of juvenile hormone-III synthase (*jhamt*) was merely increased in the high PRA treatment. Specifically, gene *cyp15a1_c1* catalyzed the conversion of methyl farnesoate to juvenile hormone III in juvenile hormone biosynthesis, suggesting an increase in juvenile hormone III in all three treatment groups, which may further result in prolonged larval traits in *D. magna*. Furthermore, up-regulated gene *jhamt* suggested the promoted production of juvenile hormone III. This may account for the reduced body length of *D. magna* in PRA treatment groups (Figure 2a). In addition, the up-regulated *jhamt* may also catalyze the conversion of farnesoic acid to generate more methyl farnesoate, which plays an important role in crustaceans’ sexual maturation by increasing the production of vitellogenin, eggs and neonates [10,37,38,39,40], as well as by stimulating gonadal development and maturation [10]. Thus, the increased methyl farnesoate may accelerate the ovarian development and promote egg maturation; in this case, *D. magna* may adjust its reproductive strategy to produce more newborns under high level of PRA stress (Figure 2b). As for the ecdysone, 26-hydroxylase (*cyp18a1*) required for the metabolic inactivation of ecdysone in *D. magna* was down-regulated only in the high PRA treatment group (Figure S4). Thus, the ecdysone level might have been slightly enhanced, whereas a significant increase (*p* < 0.05) in molting number was not detected (Figure 2c, Figure S5).

### 4.3. Genes Related to Energy Metabolism

Protein is used for maintaining homeostasis in organisms; its digestion and absorption is the main metabolic pathway of growth and reproduction [41]. In the present study, two genes involved in pancreatic secretion, including trypsin (*prss1_2_3*) and pancreatic elastase II (*cela2*), were all down-regulated in the high PRA treatment group. Gene *prss1_2_3* is involved in the formation of hydrolases that hydrolyze the corresponding protein molecules [42]. Gene *cela2* enhances insulin signaling and might have a physiological role in cellular glucose metabolism [43]. Thus, down-regulation of *cela2* and *prss1_2_3* may inhibit the hydrolysis of protein molecules and the secretion of insulin in *D. magna*.

Furthermore, down-regulated genes, including collagen type I alpha (*col1a*), collagen type IV alpha (*col4a*), and collagen type V/XI/XXIV/XXVII alpha (*col5as*) involved in fermentation by colonic bacteria process in *D. magna* were down-regulated in the high PRA treatment group. These genes are trigger factors that govern the synthesis of collagen, which is a major component for the extracellular matrix. Extracellular matrix proteins play a vital role in the development and differentiation of the gut [44], especially for the epithelial cells of the small intestine where colonic bacteria live [44,45]. Thus, the PRA-caused downregulation of *col1a*, *col4a*, and *col5a* may negatively affect the surroundings of colonic bacteria via decreasing extracellular matrix proteins in *D. magna*. Hence, the decrease in the number of colonic bacteria by PRA exposure may reduce the energy intake through fermentation in *D. magna*, which is probably further linked to the decreased body length and altered reproductive strategy.

## 5. Conclusions

To sum up, chronic exposure to PRA at all treatment groups remarkably reduced the body length of *D. magna*, and the number of newborns increased in the high PRA group. Transcriptomic analyses unraveled that the enrichment of insect hormone biosynthesis was attributed to the up-regulated genes *cyp15a1_c1* and *jhamt*, which may promote the juvenile hormone III synthesis in *D. magna* and reduce the body length. In addition, the elevated number of offspring may be due to the potentially increased methyl farnesoate suggested by the up-regulated *cyp15a1_c1*. Under the stress of PRA, *D. magna* up-regulated the genes involved in xenobiotic metabolism and inhibited the energy intake by lowering the expression of genes related to protein digestion and absorption process. Thus, the limited energy tended to be allocated to the detoxification process and to survival instead of to normal growth. As the reduced body length and the increased number of offspring in *D. magna* have been frequently reported, the toxic mechanism revealed in this study may apply to other chemicals with similar phenotypic effects.

## Figures and Tables

**Table 1:** List of crucial biological pathways disrupted by PRA exposure (*p* < 0.05).

Category	Pathway	*p* Value	Up-Gene	Down-Gene
	C-vs-L			
Metabolism	Glutathione metabolism	1.6 × 10^−3^	*gclc, gss, gst*	*-*
Metabolism	Insect hormone biosynthesis	4.4 × 10^−3^	*cyp15a1_c1, jhamt*	*-*
Metabolism	Drug metabolism—cytochrome P450	4.0 × 10^−4^	*hpgds, ugt,*	*-*
Metabolism	Drug metabolism—other enzymes	3.4 × 10^−2^	*ugt*	*-*
Metabolism	Metabolism of xenobiotics by cytochrome P450	4.9 × 10^−4^	*gst, ugt*	*-*
	C-vs-M			
Metabolism	Insect hormone biosynthesis	8.5 × 10^−3^	*cyp15a1_c1, jhamt*	*-*
	C-vs-H			
Metabolism	Glutathione metabolism	5.8 × 10^−3^	*gclc, ggt1_5, gshb, gst*	*anpep*
Metabolism	Insect hormone biosynthesis	5.2 × 10^−4^	*cyp15a1_c1, jhamt*	*cyp18a1*
Organismal Systems	Pancreatic secretion	2.7 × 10^−3^	*-*	*prss1_2_3, cela2, clca2*
Organismal Systems	Protein digestion and absorption	6.36 × 10^−7^	*-*	*cela2, col1a, col4a, col5a,* *prss1_2_3* *,*

## Data Availability

Raw data files are available through a request to the corresponding author.

## Supplementary Materials

The following supporting information can be downloaded at: https://www.mdpi.com/article/10.3390/toxics10030110/s1, Table S1: List of basic pathways affected by pravastatin sodium exposure (*p* < 0.05); Figure S1: Volcano plots of all differentially expressed genes (DEGs) in the pravastatin sodium-treated groups. The X-axis depicts the log2 fold change of DEGs and the Y-axis depicts the −log10 (adjust *p*-value); Figure S2: The correlation between fold changes (pravastatin sodium-treated groups relative to control) in mRNA levels determined by qRT-PCR and RNA-seq of genes *cyp15a1_c1, gst and dhrs4*; Figure S3: GO enrichment analysis of differentially expressed genes (DEGs) in three pravastatin sodium treated groups; Figure S4: The result of insect hormone biosynthesis pathway by transcriptomics after exposure to PRA of different concentrations for 21 days; Figure S5: Photographs of *D. magna* at 21 days.

## References

[1] Zhou Q., Liao J.K. (2009). Statins and cardiovascular diseases: From cholesterol lowering to pleiotropy. Curr. Pharm. Des..

[2] Yamashita S., Tsubakio-Yamamoto K., Ohama T., Nakagawa-Toyama Y., Nishida M. (2010). Molecular mechanisms of HDL-cholesterol elevation by statins and its effects on HDL functions. J. Atheroscler. Thromb..

[3] Hague W.E., Simes J., Kirby A., Keech A.C., White H.D., Hunt D., Nestel P.J., Colquhoun D.M., Pater H., Stewart R.A. (2016). Long-term effectiveness and safety of pravastatin in patients with coronary heart disease: Sixteen years of follow-up of the LIPID study. Circulation.

[4] Wishart D.S., Knox C., Guo A.C., Shrivastava S., Hassanali M., Stothard P., Chang Z., Woolsey J. (2006). Drugbank: A comprehensive resource for in silico drug discovery and exploration. Nucleic Acids Res..

[5] Ellesat K.S., Tollefsen K.-E., Åsberg A., Thomas K.V., Hylland K. (2010). Cytotoxicity of atorvastatin and simvastatin on primary rainbow trout (*Oncorhynchus Mykiss*) hepatocytes. Toxicol. In Vitro.

[6] Sulaiman S., Khamis M., Karaman R. (2015). Stability and removal of several statins from wastewater. Environ. Technol..

[7] Liu Y., Ding R., Pan B., Wang L., Liu S., Nie X. (2019). Simvastatin affects the expression of detoxification-related genes and enzymes in *Daphnia magna* and alter its life history parameters. Ecotoxicol. Environ. Saf..

[8] Santos M.M., Ruivo R., Lopes-Marques M., Torres T., Carmen B., Castro L.F.C., Neuparth T. (2016). Statins: An undesirable class of aquatic contaminants?. Aquat. Toxicol..

[9] Key P.B., Hoguet J., Reed L.A., Chung K.W., Fulton M.H. (2008). Effects of the statin antihyperlipidemic agent simvastatin on grass shrimp, *Palaemonetes pugio*. Environ. Toxicol. Int. J..

[10] Neuparth T., Martins C., Carmen B., Costa M.H., Martins I., Costa P.M., Santos M.M. (2014). Hypocholesterolaemic pharmaceutical simvastatin disrupts reproduction and population growth of the amphipod *Gammarus locusta* at the ng/L range. Aquat. Toxicol..

[11] Ribeiro S., Torres T., Martins R., Santos M.M. (2015). Toxicity screening of diclofenac, propranolol, sertraline and simvastatin using Danio rerio and *Paracentrotus lividus* embryo bioassays. Ecotoxicol. Environ. Saf..

[12] Dussault È.B., Balakrishnan V.K., Sverko E., Solomon K.R., Sibley P.K. (2008). Toxicity of human pharmaceuticals and personal care products to benthic invertebrates. Environ. Toxicol. Chem. Int. J..

[13] Costa A.P., Silva D.A., Rodrigues A.C., Marques C.R., Soares A.M., Rocha R.J. (2021). Species-specific oxidative stress responses and cellular energy allocation after coral shipping. Aquac. Rep..

[14] Barata C., Baird D.J. (2000). Determining the ecotoxicological mode of action of chemicals from measurements made on individuals: Results from instar-based tests with *Daphnia magna* Straus. Aquat. Toxicol..

[15] OECD (2004). Test No. 202: Daphnia sp. Acute Immobilisation Test, OECD Guidelines for the Testing of Chemicals.

[16] Pérez S., Beiras R. (2010). The mysid Siriella Armata as a model organism in marine ecotoxicology: Comparative acute toxicity sensitivity with *Daphnia magna*. Ecotoxicology.

[17] Castro B.B., Freches A., Rodrigues M., Nunes B., Antunes S. (2018). Transgenerational effects of toxicants: An extension of the daphnia 21-day chronic assay?. Arch. Environ. Contam. Toxicol..

[18] Kim H.J., Koedrith P., Seo Y.R. (2015). Ecotoxicogenomic approaches for understanding molecular mechanisms of environmental chemical toxicity using aquatic invertebrate, Daphnia model organism. Int. J. Mol. Sci..

[19] Lee B.-Y., Choi B.-S., Kim M.-S., Park J.C., Jeong C.-B., Han J., Lee J.-S. (2019). The genome of the freshwater water flea *Daphnia magna*: A potential use for freshwater molecular ecotoxicology. Aquat. Toxicol..

[20] OECD (2012). Test No. 211: Daphnia Magna Reproduction Test, OECD Guidelines for the Testing of Chemicals.

[21] Martin M. (2011). Cutadapt removes adapter sequences from high-throughput sequencing reads. EMBnet J..

[22] Kim D., Paggi J.M., Park C., Bennett C., Salzberg S.L. (2019). Graph-based genome alignment and genotyping with HISAT2 and HISAT-genotype. Nat. Biotechnol..

[23] Anders S., Pyl P.T., Huber W. (2015). HTSeq—A Python framework to work with high-throughput sequencing data. Bioinformatics.

[24] Li B., Dewey C.N. (2011). RSEM: Accurate transcript quantification from RNA-Seq data with or without a reference genome. BMC Bioinform..

[25] Yuan S., Li H., Dang Y., Liu C. (2018). Effects of triphenyl phosphate on growth, reproduction and transcription of genes of *Daphnia magna*. Aquat. Toxicol..

[26] Li J., Li H., Lin D., Li M., Wang Q., Xie S., Zhang Y., Liu F. (2021). Effects of butyl benzyl phthalate exposure on *Daphnia magna* growth, reproduction, embryonic development and transcriptomic responses. J. Hazard. Mater..

[27] Xu C., Li C.Y.-T., Kong A.-N.T. (2005). Induction of phase I, II and III drug metabolism/transport by xenobiotics. Arch. Pharmacal Res..

[28] Szakács G., Váradi A., Özvegy-Laczka C., Sarkadi B. (2008). The role of ABC transporters in drug absorption, distribution, metabolism, excretion and toxicity (ADME–Tox). Drug Discov. Today.

[29] Halliwell B., Gutteridge J.M. (2015). Free Radicals in Biology and Medicine.

[30] Bhabak K.P., Mugesh G. (2010). Functional mimics of glutathione peroxidase: Bioinspired synthetic antioxidants. Acc. Chem. Res..

[31] Hayes J.D., Flanagan J.U., Jowsey I.R. (2005). Glutathione transferases. Annu. Rev. Pharmacol. Toxicol..

[32] Hiruma K., Kaneko K. (2013). Hormonal regulation of insect metamorphosis with special reference to juvenile hormone biosynthesis. Curr. Top. Dev. Biol..

[33] Miyakawa H., Sato T., Song Y., Tollefsen K.E., Iguchi T. (2018). Ecdysteroid and juvenile hormone biosynthesis, receptors and their signaling in the freshwater microcrustacean Daphnia. J. Steroid Biochem. Mol. Biol..

[34] Riddiford L.M. (2012). How does juvenile hormone control insect metamorphosis and reproduction?. Gen. Comp. Endocrinol..

[35] Liu S., Li K., Gao Y., Liu X., Chen W., Ge W., Feng Q., Palli S.R., Li S. (2018). Antagonistic actions of juvenile hormone and 20-hydroxyecdysone within the ring gland determine developmental transitions in Drosophila. Proc. Natl. Acad. Sci. USA.

[36] Miyakawa H., Toyota K., Hirakawa I., Ogino Y., Miyagawa S., Oda S., Tatarazako N., Miura T., Colbourne J.K., Iguchi T. (2013). A mutation in the receptor Methoprene-tolerant alters juvenile hormone response in insects and crustaceans. Nat. Commun..

[37] Laufer H., Biggers W.J. (2001). Unifying concepts learned from methyl farnesoate for invertebrate reproduction and post-embryonic development. Am. Zool..

[38] Reddy P.R., Nagaraju G.P.C., Reddy P.S. (2004). Involvement of methyl farnesoate in the regulation of molting and reproduction in the freshwater crab *Oziotelphusa senex*. J. Crustacean Biol..

[39] Mak A.S.C., Choi C.L., Tiu S.H.K., Hui J.H.L., He J.G., Tobe S.S., Chan S.M. (2005). Vitellogenesis in the red crab Charybdis Feriatus: Hepatopancreas-specific expression and farnesoic acid stimulation of vitellogenin gene expression. Mol. Reprod. Dev. Inc. Gamete Res..

[40] LeBlanc G.A. (2007). Crustacean endocrine toxicology: A review. Ecotoxicology.

[41] Wang P., Ng Q.X., Zhang H., Zhang B., Ong C.N., He Y. (2018). Metabolite changes behind faster growth and less reproduction of *Daphnia similis* exposed to low-dose silver nanoparticles. Ecotoxicol. Environ. Saf..

[42] Schilling O., Biniossek M.L., Mayer B., Elsässer B., Brandstetter H., Goettig P., Stenman U.-H., Koistinen H. (2018). Specificity profiling of human trypsin-isoenzymes. Biol. Chem..

[43] Esteghamat F., Broughton J.S., Smith E., Cardone R., Tyagi T., Guerra M., Szabó A., Ugwu N., Mani M.V., Azari B. (2019). CELA2A mutations predispose to early-onset atherosclerosis and metabolic syndrome and affect plasma insulin and platelet activation. Nat. Genet..

[44] Ratcliffe D.R., Iqbal J., Hussain M.M., Cramer E.B. (2009). Fibrillar collagen type I stimulation of apolipoprotein B secretion in Caco-2 cells is mediated by β1 integrin. Biochim. et Biophys. Acta (BBA)-Mol. Cell Biol. Lipids.

[45] Basson M.D. (2003). Invited research review: Cell-matrix interactions in the gut epithelium. Surgery.

