# Supplementary material for: Transcriptomic Alterations in Water Flea (Daphnia magna) following Pravastatin Treatments: Insect Hormone Biosynthesis and Energy Metabolism
Source: Toxics. 2022 Feb 25;10(3):110. doi: 10.3390/toxics10030110 (PMC8952691; doi:10.3390/toxics10030110)
Supplement: Supplementary file 1 [file toxics-10-00110-s001.zip › toxics-1603861-supplementary.pdf]

toxics

MDPI

Supplementary Materials: Transcriptomic Alterations in Water Flea (*Daphnia magna*) Following Pravastatin Treatments: Insect Hormone Biosynthesis and Energy Metabolism

Yuan Lei, Jiahua Guo, Qiqi Chen, Jiezhang Mo, Yulu Tian, Hisato Iwata and Jinxi Song

Table S1. List of basic pathways affected by pravastatin sodium exposure ($p < 0.05$).

Category	Pathway	*P* value	Up-gene	Down-gene
	C-vs-L			
Human Diseases	Amphetamine addiction	7.0×10^{-3}	*ddc*	-
Human Diseases	Chemical carcinogenesis	3.9×10^{-5}	*cox2, gst, ugt*	-
Human Diseases	Cocaine addiction	3.8×10^{-3}	*ddc*	-
Metabolism	Arachidonic acid metabolism	6.3×10^{-3}	*gst*	-
Metabolism	Arginine biosynthesis	4.3×10^{-2}	*nos1*	-
Metabolism	Ascorbate and aldarate metabolism	4.4×10^{-3}	*ugt*	-
Metabolism	Cysteine and methionine metabolism	7.0×10^{-3}	*gclc, gss*	-
Metabolism	Pentose and glucuronate interconversions	2.6×10^{-4}	*akr1b, ugt*	-
Metabolism	Phenylalanine metabolism	1.4×10^{-3}	*ddc*	-
Metabolism	Porphyrin and chlorophyll metabolism	7.7×10^{-3}	*ugt*	-
Metabolism	Retinol metabolism	1.3×10^{-2}	*ugt*	-
Metabolism	Starch and sucrose metabolism	1.3×10^{-2}	*amy*	-
Metabolism	Steroid hormone biosynthesis	8.1×10^{-3}	*ugt*	-
Metabolism	Tryptophan metabolism	5.3×10^{-3}	*ddc*	-
Metabolism	Tyrosine metabolism	1.5×10^{-3}	*ddc*	-
Organismal Systems	Carbohydrate digestion and absorption	8.9×10^{-3}	*amy*	-
Organismal Systems	Dopaminergic synapse	1.3×10^{-2}	*ddc*	-
Organismal Systems	Serotonergic synapse	3.3×10^{-4}	*cox2, ddc*	-
	C-vs-M			
Cellular Processes	Lysosome	2.9×10^{-2}	*npc2*	*cd63, ctsl*
Environmental Information Processing	FoxO signaling pathway	4.1×10^{-2}	-	*fbxo25_32, g6pc*
Human Diseases	Proteoglycans in cancer	1.9×10^{-2}	*twist*	*cd63, ctsl*
	C-vs-H			
Cellular Processes	Ferroptosis	2.5×10^{-2}	*fth1, gclc*	-
Environmental Information Processing	ECM-receptor interaction	3.9×10^{-2}	-	*col1a, col4a*
Environmental Information Processing	Neuroactive ligand-receptor interaction	6.8×10^{-3}	-	*gria1, prss1_2_3*
Human Diseases	Chemical carcinogenesis	2.5×10^{-3}	*cyp1a1, gst, ugt,*	-
Human Diseases	Influenza A	3.0×10^{-2}	*prss1_2_3*	-
Human Diseases	Staphylococcus aureus infection	1.0×10^{-2}	*cfd*	*cyp3a*
Metabolism	Amino sugar and nucleotide sugar metabolism	6.7×10^{-3}	*e3.2.1.14*	*anpep*
Metabolism	Glycosaminoglycan biosynthesis - chondroitin sulfate / dermatan sulfate	2.5×10^{-2}	*csgalnact1_2, ust*	-
Metabolism	Linoleic acid metabolism	1.0×10^{-2}	*cyp3a*	*grik2, prss1_2_3*

Metabolism	Monobactam biosynthesis	4.6×10^{-5}	-	*cela2, col1a, col4a, prss1_2_3,*
Metabolism	Retinol metabolism	1.2×10^{-3}	*cyp3a4, ugt*	*dhrs3, dhrs4, rdh5, rdh8*
Metabolism	Selenocompound metabolism	4.4×10^{-3}	-	*cela2, clca2, prss1_2_3*
Metabolism	Starch and sucrose metabolism	3.2×10^{-2}	*amy, e3.2.1.4, tps*	*prss1_2_3*
Metabolism	Steroid hormone biosynthesis	1.4×10^{-2}	*cyp3a, ugt*	-
Metabolism	Sulfur metabolism	1.7×10^{-3}	-	*cyp3a4, dhrs3, dhrs4, rdh5, rdh8*
Organismal Systems	Antigen processing and presentation	3.6×10^{-2}	*ctss,*	*ctsb*
Organismal Systems	Complement and coagulation cascades	1.7×10^{-3}	*cfd*	*papss*

C-vs-L

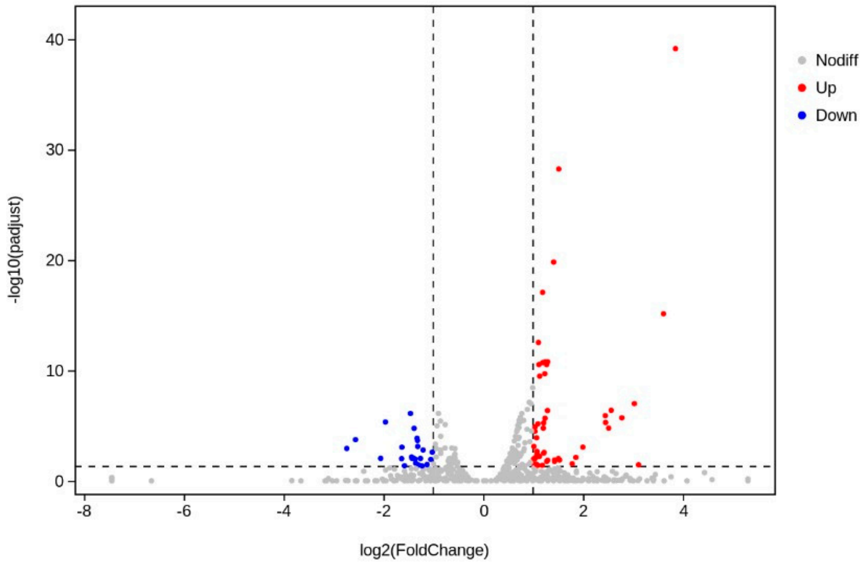

C-vs-M

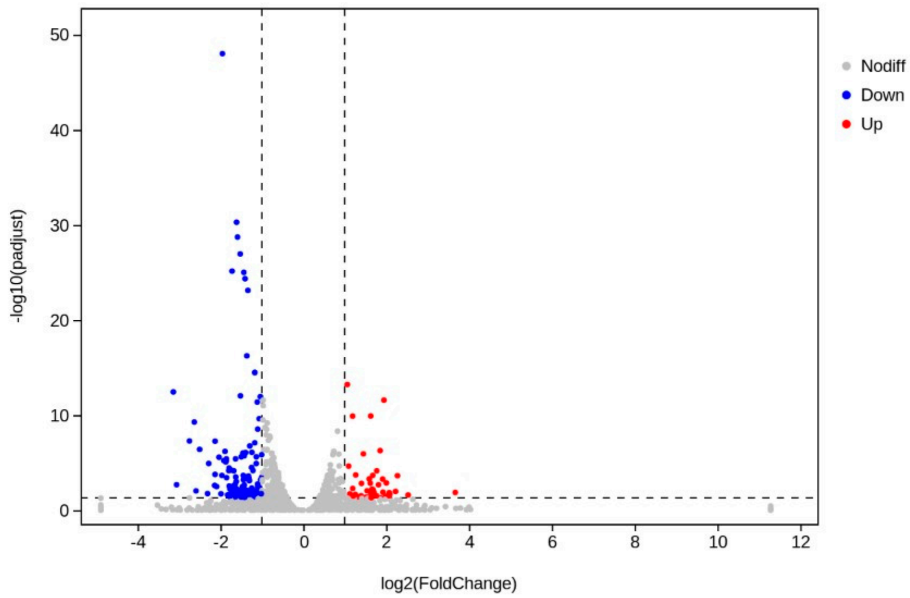

C-vs-H

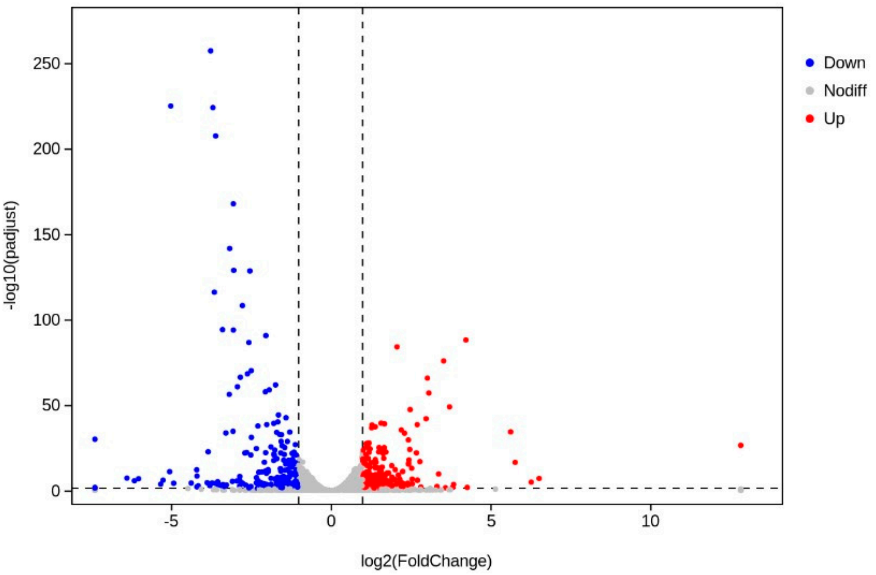

Figure S1. Volcano plots of all differentially expressed genes (DEGs) in the pravastatin sodium-treated groups. The X-axis depicts the log2 fold change of DEGs and the Y-axis depicts the –log 10 (adjust p value).

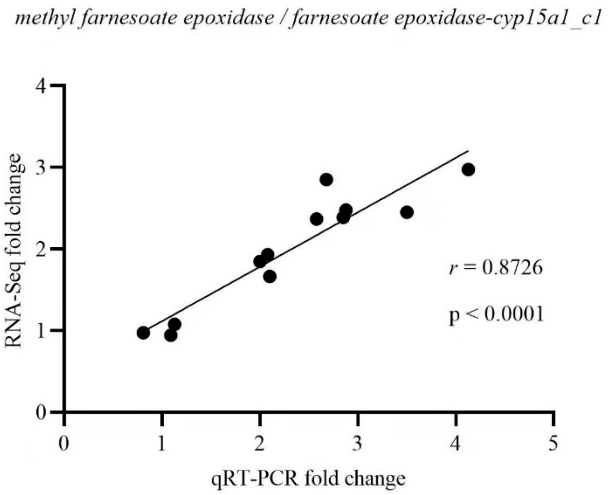

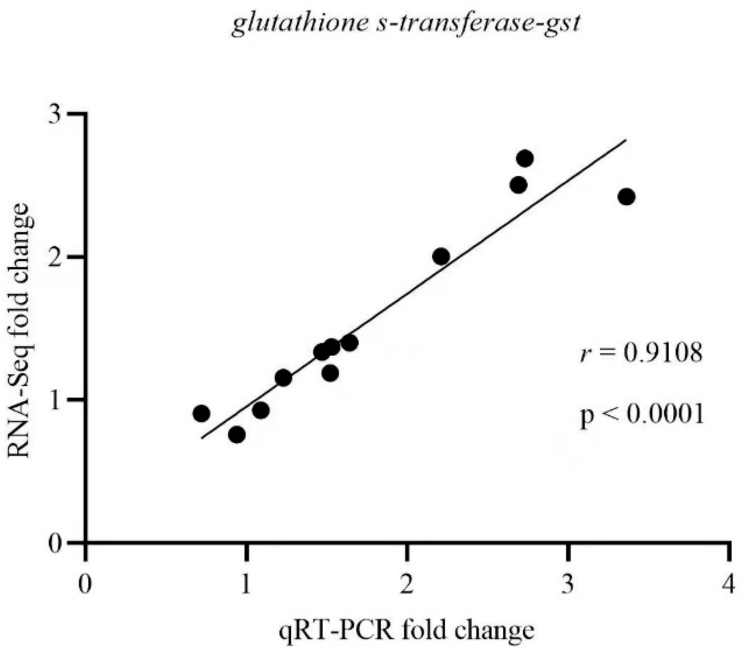

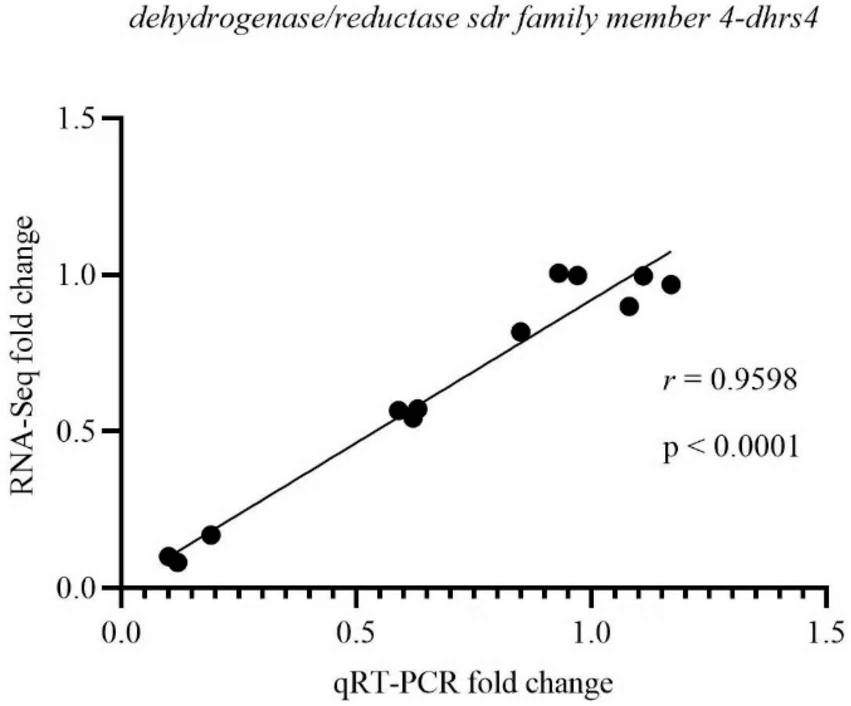

Figure S2. The correlation between fold changes (pravastatin sodium-treated groups relative to control) in mRNA levels determined by qRT-PCR and RNA-seq of genes *cyp15a1_c1, gst and dhrs4.*

C-vs-L

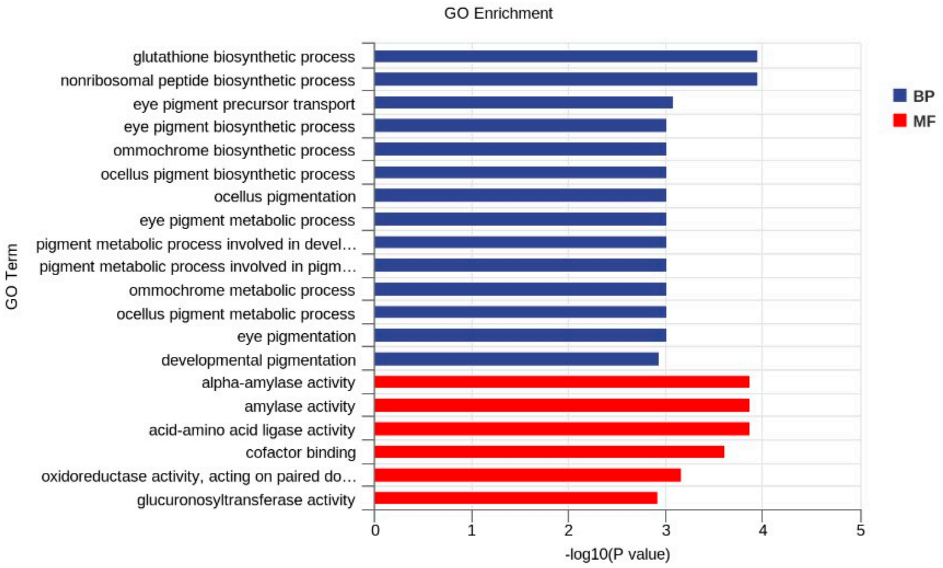
GO Enrichment
glutathione biosynthetic process
nonribosomal peptide biosynthetic process
eye pigment precursor transport
eye pigment biosynthetic process
ommochrome biosynthetic process
ocellus pigment biosynthetic process
ocellus pigmentation
eye pigment metabolic process
pigment metabolic process involved in devel…
pigment metabolic process involved in pigm…
ommochrome metabolic process
ocellus pigment metabolic process
eye pigmentation
developmental pigmentation
alpha-amylase activity
amylase activity
acid-amino acid ligase activity
cofactor binding
oxidoreductase activity, acting on paired do…
glucuronosyltransferase activity
GO Term
0
1
2
3
4
5
-log10(P value)
BP
MF

C-vs-M

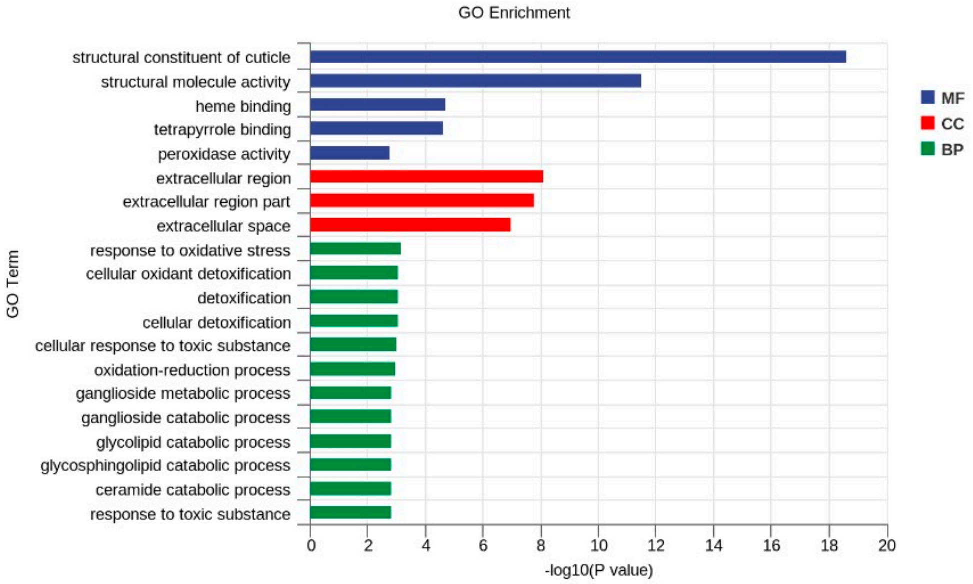
GO Enrichment
structural constituent of cuticle
structural molecule activity
heme binding
tetrapyrrole binding
peroxidase activity
extracellular region
extracellular region part
extracellular space
response to oxidative stress
cellular oxidant detoxification
detoxification
cellular detoxification
cellular response to toxic substance
oxidation-reduction process
ganglioside metabolic process
ganglioside catabolic process
glycolipid catabolic process
glycosphingolipid catabolic process
ceramide catabolic process
response to toxic substance
GO Term
0
2
4
6
8
10
12
14
16
18
20
-log10(P value)
MF
CC
BP

C-vs-H

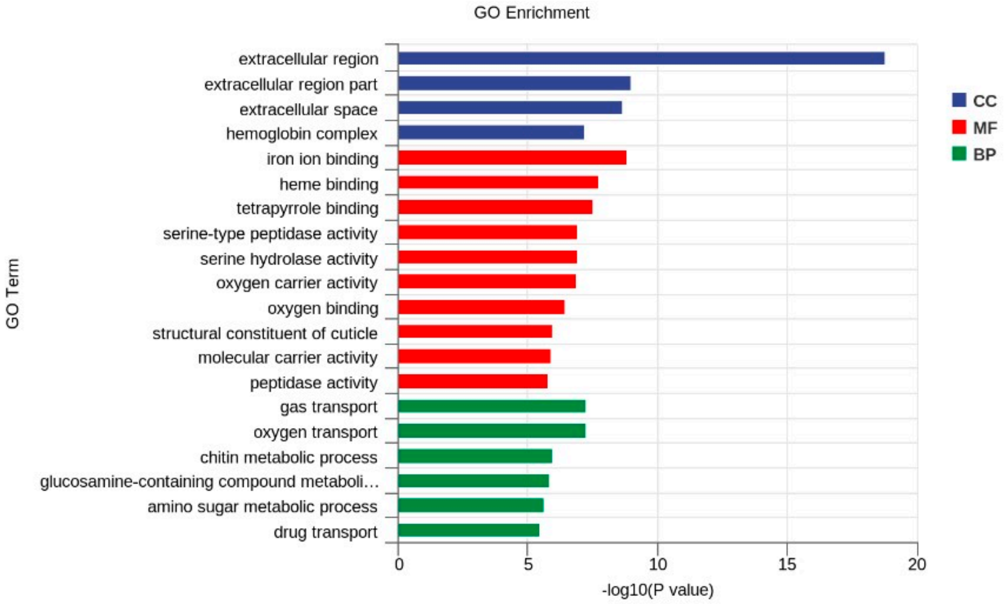

Figure S3. GO enrichment analysis of differentially expressed genes (DEGs) in three pravastatin sodium treated groups.

C-vs-L

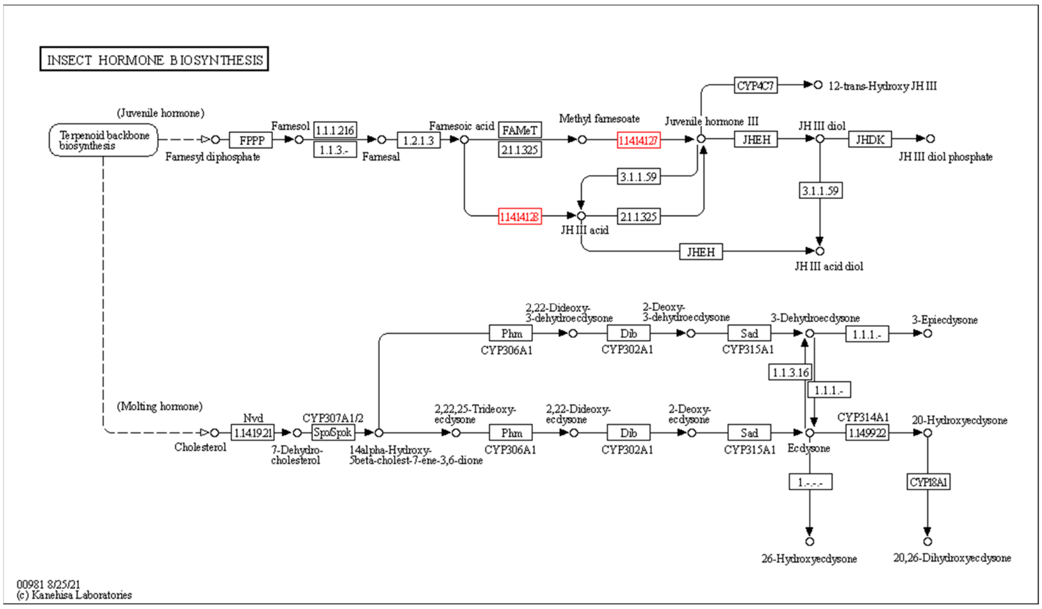

C-vs-M

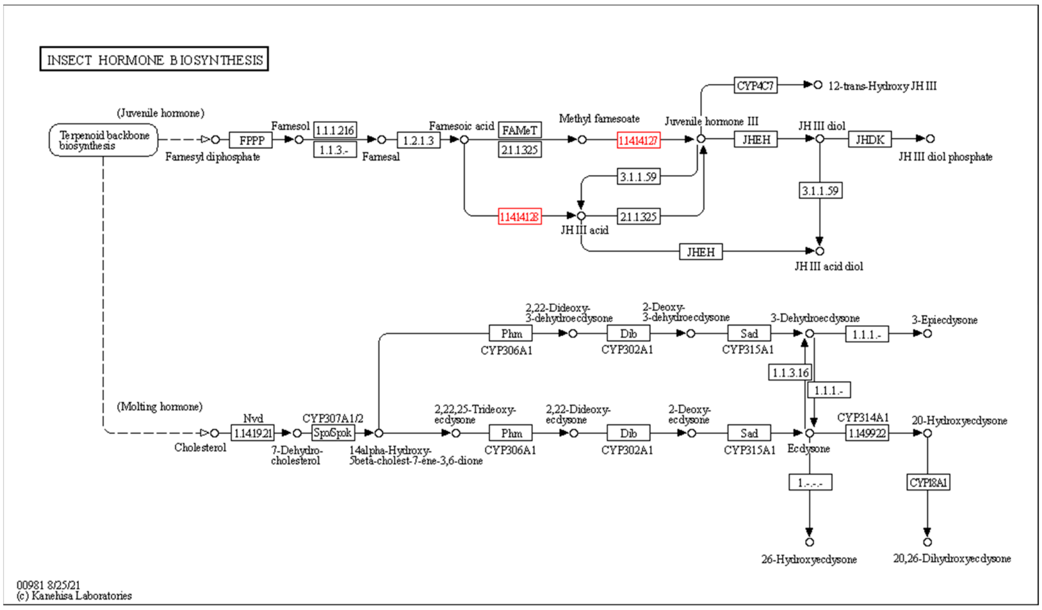

C-vs-H

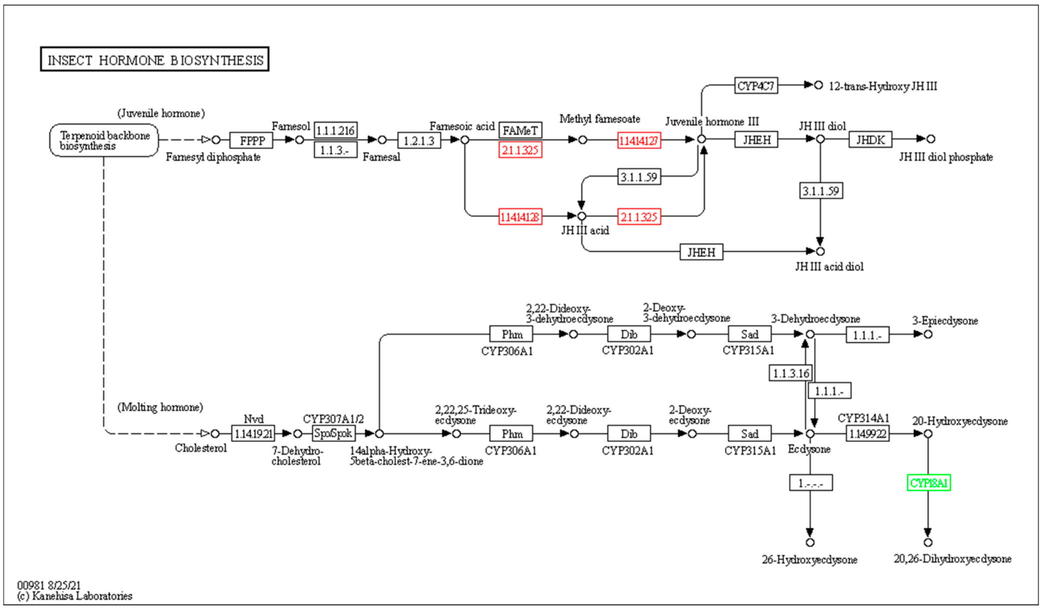

Figure S4. The result of insect hormone biosynthesis pathway by transcriptomics after exposure to PRA of different concentrations for 21 days.

Control group

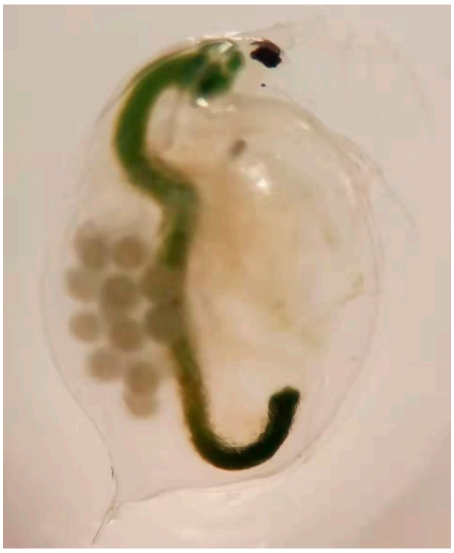

High PRA treatment group

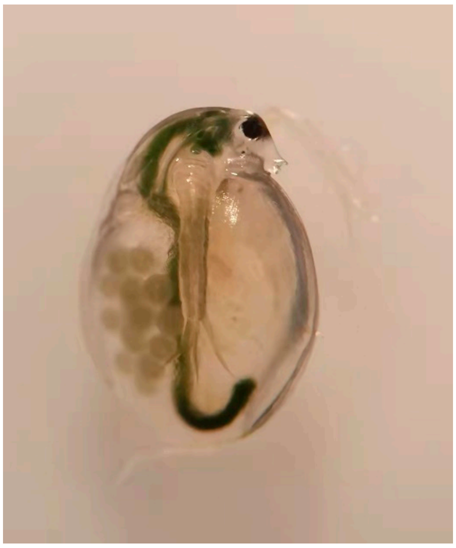

Figure S5. Photographs of *D. magna* at 21 days.
